# The Family’s Contribution to Patient Safety

**DOI:** 10.3390/nursrep13020056

**Published:** 2023-04-07

**Authors:** Tânia Correia, Maria Manuela Martins, Fernando Barroso, Lara Pinho, João Longo, Olga Valentim

**Affiliations:** 1 Instituto de Ciências Biomédicas Abel Salazar (ICBAS), Universidade do Porto (UP), 4050-313 Porto, Portugal; 2 CINTESIS@RISE, Nursing School of Porto (ESEP), 4200-450 Porto, Portugal; 3 Escola Superior de Saúde de Viseu (ESSV), Instituto Politécnico de Viseu (IPV), 3500-843 Viseu, Portugal; 4 Escola Superior de Enfermagem do Porto (ESEP), 4050-313 Porto, Portugal; 5 Centro Hospitalar de Setúbal, 2910-446 Setúbal, Portugal; 6 Nursing Department, Universidade de Évora, 7000-811 Évora, Portugal; 7 Comprehensive Health Research Centre (CHRC), Universidade de Évora, 7000-811 Évora, Portugal; 8 Nursing Research, Innovation and Development Centre of Lisbon (CIDNUR), 1990-096 Lisboa, Portugal; 9 Escola Superior de Saúde Ribeiro Sanches (ERISA)–IPLUSO, 1950-396 Lisboa, Portugal; 10 Escola Superior de Enfermagem de Lisboa (ESEL), 1600-096 Lisboa, Portugal

**Keywords:** family nursing, family-centered care, hospitalization, patient safety, safety management

## Abstract

Background: Person- and family-centered care is one of the recommendations to achieve quality of care and patient safety. However, many health professionals associate the family with insecurity in care. Objective: To analyze, based on nurses’ statements, the advantages and disadvantages of the family’s presence in hospitals for the safety of hospitalized patients. Methods: This was a qualitative interpretative study based on James Reason’s risk model, conducted through semi-structured interviews with 10 nurses selected by convenience. A content analysis was performed using Bardin’s methodology and MAXQDA Plus 2022 software. Results: We identified 17 categories grouped according to the representation of the family in patient safety: The family as a Potentiator of Security Failures (7) and Family as a Safety Barrier (10). Conclusions: The higher number of categories identified under Family as a Safety Barrier shows that nurses see strong potential in the family’s involvement in patient safety. By identifying the need to intervene with and for families so that their involvement is safe, we observed an increase in the complexity of nursing care, which suggests the need to improve nursing ratios, according to the participants.

## 1. Introduction

Patient safety is currently an important global public health concern. The World Health Organization (WHO) estimates that there are 421 million hospitalizations annually in the world, and about 42.7 million adverse events occur with patients during these hospitalizations. In total, 50% of these adverse events are preventable. In high-income countries, about 1 in 10 patients are affected. These damages caused to patients are considered the 14th cause of morbidity and mortality in the world. These damages result in a high cost, representing 15% of hospital activity and expenditure in Organization for Economic Cooperation and Development (OECD) countries [1,2].

Therefore, the provision of safe health care, in addition to representing a challenge, is a fundamental element in the quality of care and an undoubted right of patients [3]. According to the WHO, “safety is the reduction of risk of unnecessary harm to an acceptable minimum. An acceptable minimum refers to the collective notions of given current knowledge, re-sources available and the context in which care was delivered weighed against the risk of non-treatment or other treatment” [4].

**Figure 1:**
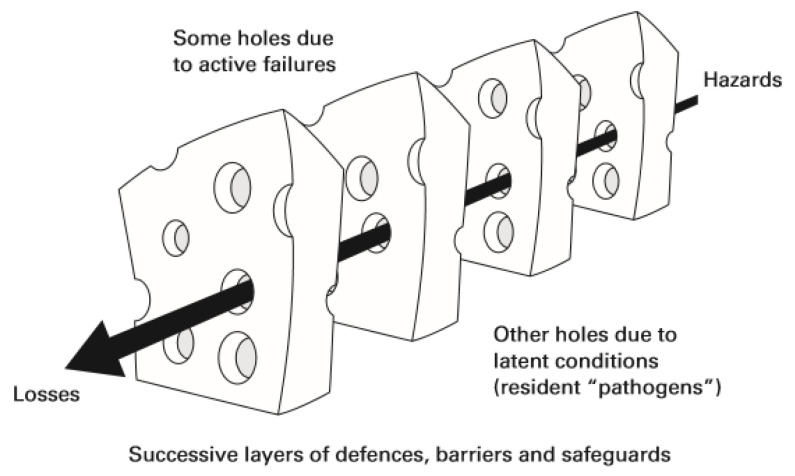
James Reason’s Swiss cheese model. Source: Reason et al. [6].

Both because of its alarming effects on health safety and because of its complexity, it is essential to analyze the mechanisms behind error causality. James Reason’s Swiss cheese model (Figure 1) explains the dynamics of failure in an organization. It considers that systems have barriers (e.g., equipment, people, or technologies) that are strategically positioned to avoid errors (slices of cheese) [5].

Ideally, these barriers should not have flaws; however, the model admits the existence of flaws, representing them as holes in the Swiss cheese slices/barriers. It characterizes faults as dynamic, in that the holes open and close in different locations. The occurrence of isolated faults does not necessarily imply the occurrence of an error. This arises when failures in all the barriers are aligned in a risk trajectory for patient safety. This model makes it possible to contribute to a more proactive form of risk management by identifying flaws, risks, and potential strategic barriers [5,6].

Among the recommended strategies for improving patient safety is the adoption of person- and family-centered care [7]. With the publication of the Institute of Medicine’s report, *Crossing the Quality Chasm: A New Health System for the 21st Century*, person- and family-centered care has gained greater recognition [8]. Thus, the Person and Family Centered Care model (PFCC) is established as fundamental to improving health care in terms of communication, patient satisfaction, and safety.

Patient and Family-Centered Care (PFCC) is a care model that is based on mutually beneficial partnerships between health-care providers, patients, and families in various health-care contexts [9]. Patients and family members, in collaboration with health teams, define and establish who participates in the care process, namely, in decision-making, and how they do so [9].

Evidence has emerged that the PFCC contributes to quality and patient safety. Concretely, it leads to better health outcomes, such as the reduction in hospitalizations and health errors, improvements in the care experience and the satisfaction of patients and families, and more effective management of resources [9,10,11].

The concept of the family has evolved and is mostly represented in a biological, social, psychological, and legal perspective. In the context of health care and nursing, the family is defined as a group that is characterized by being a social unit or collective whole formed by people united through consanguinity, affinity, and emotional and/or legal relationships, and which is considered greater than the sum of its parts [12]. However, the more theoretical references state that the family comprises two or more people who recognize each other as a family and depend on each other in terms of emotional, physical, and economic support [13]. Wright and Lorraine reinforce the notion that the concept of family implies self-recognition: the family is who the person identifies as such [14].

Nurses recognize partnership with families as a contribution to improving quality of care, although it does not translate into the practice of care [15,16]. These professionals also see families as risks to patient safety, as hindering the care process, and as sources of an increase in workload [15,17].

Thus, this study aims to analyze, based on nurses’ statements, the advantages and disadvantages of the family’s presence in hospitals for the safety of hospitalized patients.

## 2. Materials and Methods

This study focuses on a complex, dynamic, multifactorial phenomenon and, considering the scarcity of studies in this area, this is a qualitative, interpretive study, based on James Reason’s Swiss cheese risk model [6].

An intentional non-probabilistic sampling method was used. The participants in this study were nurses in 3 Portuguese hospitals in the North, selected for convenience, as they were the most accessible and simultaneously met the inclusion criteria. These criteria were: at least 4 years of experience (Benner considers 3 to 5 years of experience for expertise [18]), experience in hospital inpatient services for adults, exercising duties at the time of the interview, and availability to participate. In the eighth and subsequent interviews, no new data/categories were found, so in the tenth interview, we considered that data saturation was reached.

Of the total of 10 participants, 9 were female and 1 was male; the participants were aged between 28 and 62 years, and with between 6.5 and 39 years of experience. Seven participants had Masters degrees and nine were specialists (in rehabilitation nursing (6), in medical-surgical nursing (2), and in mental-health and psychiatric nursing (1)).

Data collection was carried out using individual semi-structured interviews with open questions, which allowed better understanding of the participants’ points of view, and a more explicit idea of their experiences [19]. The interviews took place between June and September 2020 via Zoom. The questions for which data were analyzed in this phase were:

- What assessment do you make of the presence of the family with hospitalized patients?
- How do you consider the family for the safety of care?

After transcribing the interviews, they were sent to the respective interviewees for validation.

Content analysis was carried out according to the three phases of Bardin’s methodology: pre-analysis, material exploration, and treatment of obtained results/interpretation [20].

Common contents were sought in exploratory reading to identify thematic areas, called categories. The James Reason Model [5,6]) was the theoretical framework used in this process. The MAXQDA Plus 2022 software was used as a tool to help codify and categorize the interviews. Subsequently, the units of analysis were grouped into large thematic areas (families). In this categorization process, we considered the semantic criterion.

We declare that this report adhered to the COREQ guidelines for qualitative-research reporting [21].

Ethical and legal principles were respected. The present study was authorized by the joint Ethics Committee of the Oporto Hospital and University Center and the Abel Salazar Biomedical Sciences Institute (ICBAS) of the Oporto University (UP). In accordance with the Declaration of Helsinki, anonymity, confidentiality, and informed consent were respected in all procedures performed with the participants.

The data include information which allows the identification of hospitals, services, and nurses participating in the study. Out of respect for confidentiality, data beyond those presented in the study are not available.

Due to the nature of the study, sample size, sampling methodology, and the origin of the participants in a single country, limitations as to the generalizability of the results presented are acknowledged.

## 3. Results

**Figure 2:**
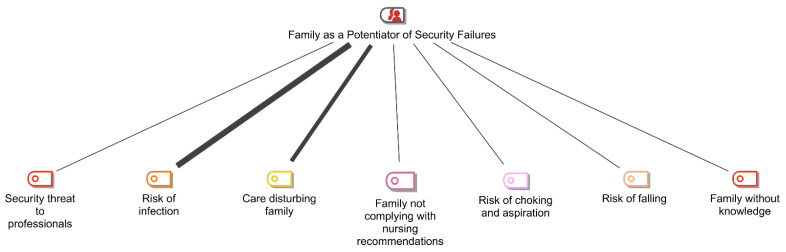
Items identified in the category of “Family as a Potentiator of Security Failures”. Source: MAXQDA Plus 2022.

**Figure 3:**
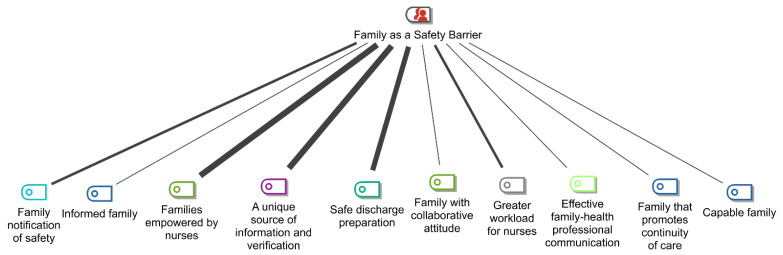
Items identified in the category: “Family as a Safety Barrier”. Source: MAXQDA Plus 2022.

In the content analysis of the interviews with the participating nurses, 17 categories were identified. These were grouped into two major thematic areas (families) according to the positioning of these categories in James Reason [6]’s risk model (Figure 2): *Family as a Potentiator of Security Failures* and *Family as a Security Barrier*. Figure 2 and Figure 3 show the respective items in each category, and their connections vary in thickness according to the frequency with which they were identified in the analysis of the interviews. The thicker the connection, the higher its frequency.

Within the scope of the Family as Potentiator of Security Failures, seven categories were identified: security threat to professionals, risk of infection, care-disturbing family, family not complying with nursing recommendations, risk of choking and aspiration, risk of falling, and family without knowledge. It appears that the categories with the highest level of identification in the nurses’ speeches were risk of infection and family-disturbing care.

Ten categories were identified within the scope of Family as a Safety Barrier (Figure 3): family notification of safety events, informed family, families empowered by nurses, a unique source of information and verification, safe-discharge preparation, family with collaborative attitude, greater workload for nurses, effective family–health-professional communication, family that promotes continuity of care, and capable family.

The most frequently identified categories were: families empowered by nurses, a unique source of information and verification, safe-discharge preparation, greater workload for nurses, and family notification of safety events.

## 4. Discussion

In this study, it is possible to verify, in the statements of the participating nurses, an ambivalent opinion regarding the role that the family plays in the safety of hospitalized patients. On the one hand, the family is perceived as a potential source of security failures; on the other, it is seen as a security barrier, considering James Reason’s risk model [6].

### 4.1. Family as Potentiator of Security Failures

The threat to the professionals’ safety from the family (*Security threat to professionals*) was mentioned by the participants as one of the risks of the family’s presence in the hospital: “it has happened to me, for example, a family member being a little more aggressive both verbally and physically. It was difficult for me to reach that relative and try to explain that his way of being would not be the best. So, in this case, he wasn’t available to listen either, but the strategy was to call the security guard and get him out of there” (E7). Experiences of physical violence were not reported, but there were examples of verbal violence. Violence against health professionals has become a growing and global problem [22,23]. Due to their direct contact with patients and their companions, nurses are three times more likely to experience violence [24]. Evidence shows that violence in hospitals poses a threat to the health of health professionals and patients, decreases the quality of care, reduces the concentration of nurses when performing their duties and increases the occurrence of errors, in addition to reducing the motivation and satisfaction of health professionals and causing them physical and psychological harm [23]. There is a great focus on the rights of patients; however, data on the recognition of the rights of health professionals and nurses by patients and companions are few [22].

The *risk of infection* was another of the risks associated with the family in hospitals by the participating nurses: “Families still do not have this sensitivity of arriving at the service and washing their hands, restricting themselves to the space of their sick family member” (E3). Contrary to the participants’ perceptions, previous studies demonstrate that the presence of the family in adult intensive care is not associated with increased rates of infection [25]. During the COVID-19 pandemic crisis, the Netherlands opened 26 nursing homes to family members and there were no records of new infections [26]. In fact, the evidence does not demonstrate a substantial role for visits in the transmission of COVID-19 in hospitals [27].

The family was also referred to as a potential source of security failures, in which a family disturbs care (*Care disturbing family*): “if the family is disturbing [care], it jeopardizes the safety of other users and the family member themselves” (E10. Previous data show that the presence of the family in the hospital can negatively affect the development and work environment of nurses, who often report a loss of control and interruptions in associated care [28].

In the same sense, the participants referred that families that do not comply with nursing recommendations (*family not complying with nursing recommendations*) are sources of insecurity for patients: “I think that families only jeopardize the safety of users if they do not comply with our provision of care. care. For example, I have the patient immobilized, I even explain why he is immobilized, and the family member still removes the immobilization, and the patient pulls out the nasogastric tube. This happens very frequently, and, in these situations, families question the provision of care and patient safety. I’ve also had patients who almost fell because the family had removed the safety band” (E3). Although there is evidence on violence against health professionals and the disturbances this may cause, regarding non-compliance with nursing recommendations, no data are available. However, the guidelines for promoting positive collaboration with families and preventing them from becoming sources of disturbance have supported their involvement in the care process [9,10].

The participants also associated the family with the *risk of choking and aspiration*: “…the patient had a soft diet due to difficulties in swallowing and the family thought they could give him some grapes. It put the patient’s safety at risk” (E3). The same association was made with the *risk of falling*: “we trust the relatives explaining... we lower the railing, but we always tell them, before they leave, to ring the bell or let us know they’re leaving so we can raise the railing again. And fortunately it has happened few times but unfortunately it happens, the relative has gone and the bed rail has been left down with the risks associated with that” (E8). Regarding the causes of errors and adverse health events and their associated factors, studies indicate that there are several factors involved and the most frequent is the organizational structure [29]. A study carried out in 21 Dutch hospitals revealed that 39% of adverse events had patient-related factors as their main causes. These failures are related to the patients’ characteristics or conditions, such as communication skills or compliance with treatment [30]. This finding is not clear regarding the involvement of the family in this process, and no relevant data were found about this association. However, there are data that suggest that families play an important role in patients’ adherence to treatment, which is identified as a cause of errors [31,32,33].

The category of *family without knowledge* was identified as a risk to the safety of patients: “I consider that the greatest fragility is the lack of knowledge and they end up touching the equipment, the beds, the chairs, all very risky surfaces” (E5). In this sense, training and teaching strategies and interventions for the families of hospitalized patients have shown positive effects on patient safety [11,34,35].

### 4.2. Family as a Safety Barrier

The notification of adverse events by the family (*family notification of safety*) was frequently mentioned by the participants as one of the reasons why the family represents a source of added value for the safety of patients: “I have already had a situation where a family member says that the mother is called Maria Alice and has a bracelet with another name. I thank the family for their cooperation and correct the situation” (E10). Available data indicate that when the family actively participates in the identification and reporting of adverse events, it is possible to detect and learn about adverse events that otherwise would not have been identified. This participation increases the reporting of error rates and adverse events and contributes to the improvement of hospital safety and research [36].

The participants also mentioned that an *informed family* represents a safety barrier: “Families are very informed about hand washing. There are already a lot of kittens there, people arrive at the service, if they don’t wash their hands, they use the sterillium … this is safety” (E10). This is again evident in other research results [11,34,35].

The participants strongly recognized that nurses can play a fundamental role and that families become safer if they are trained by nurses (*families empowered by nurses*): “I think that the presence of families has more advantages than disadvantages and we should be elements that promote their presence to provide the safety of hospitalized patients. We must teach so that the behavior of family members does not compromise safety in the provision of care” (E2). In this context, families report that they feel unprepared and poorly monitored by health professionals [11].

Nurses also very often mentioned that the family is a unique and irreplaceable source of information and verification in the field of health care (*a unique source of information and verification*): “The issue of anamnesis, the issue of the patient’s background, what behaviors they had at home, what were the activities in clinical terms, in terms of daily life process, this information fails when the family is not present” (E8). They also emphasized that the family relationship is what allows this specificity, which no professional can replace: “I think that users are afraid to tell us about some situations and then with the family, as they feel more comfortable, they tell us what really happens” (E2). In addition to being a very important source of information about the safety of family members, it is widely recognized and proven that each family is unique and has special expertise about patients and their needs, which gives the family a unique and irreplaceable character [36,37].

The importance of the family’s role in preparing for safe discharge (*safe-discharge preparation*) was described by the participants with conviction: “As a positive point, I consider that it is always an important foundation in the safety of the preparation process for discharge and post-hospitalization, namely in the management of the disease, the medication, nutritional regime and the like” (E7). After hospital discharge, about 50% of adults suffer from medical errors, and 19% to 23% suffer an adverse event, the most frequent of which are related to medication. The guidelines for safe discharge refer to a structured plan, which includes the family [38].

The item of *family with a collaborative attitude* was also referred to as fundamental for patients’ safety: “If the family wants to have a collaborative attitude with us in terms of learning, enriching strategies to later use in home care, I think it is extremely positive the presence of families” (E3). Effectively, studies carried out in this area demonstrate that families are motivated to participate in care, even when they do not have the necessary knowledge [11].

To guarantee the safe involvement of the family in the care process (*greater workload for nurses*), the participants mentioned with some frequency that this implies a greater workload for nurses: “We should encourage more families to participate in care. However, this entails more time expenditure, a higher ratio of nurses to be able to assist the families. We know perfectly well that when we are going to receive a family, we are going to need more time” (E3). Some studies show that nurses perceive an increase in their workload in association with the presence of the family in the hospital [28,39]. However, one study showed that the implementation of care policies centered on the person and family caused more stress in more experienced professionals, therapists, nutritionists, pharmacists, and social workers. Nurses experienced an increased workload but a lower level of stress. This is because, despite this increase in workload, there was an increase in patient and family satisfaction, with an effect on the interaction with nurses [40].

The participants described effective communication between family and health professionals as a contribution to patient safety (*effective family–health-professional communication*): “when the family is present, if we need to communicate, it is easier and a moment of health education is more easily provided… of information exchange” (E4). Communication failures in health care have been widely recognized as causes of health errors [41]. Family-centered care requires a systematic communication strategy between health-care teams and families [42]. A study on the results of implementing a structured communication intervention for family-centered rounds showed that, although general errors did not change, harmful errors decreased, and family experiences and communication processes improved [43]. However, errors associated with problems in the communication process with families require further investigation.

The family’s contribution to the continuity of care (*family that promotes continuity of care*) was also referred to as essential for patient safety, not only with regard to discharge but throughout the hospitalization process, with one participant highlighting “continuity when information is exchanged with the family about the patient’s health process” (E5). Continuity of care involves a set of health-care events that are linked and consistent with the person’s individual and health needs [44]. It involves fluidity in the transition to information, and effective communication between patients, families, and health professionals [44,45,46]. Due to the importance of this continuity and the information in question, continuity of care has a strong relationship with quality, efficiency, and safety in health [44].

Finally, the nurse participants mentioned that the family is safer when it is able to understand the information provided by nurses and collaborate (*capable family*): “In general, families contribute to the safety of patients depending on their ability to collaborate and understand the risk that the patient has” (E8). Studies show that families, although motivated to participate and collaborate in the health-care process, feel unprepared to do so [11].

Through the number of items in each of the two major categories, it is possible to verify that, according to the statements of the participants, the Family as a Safety Barrier, with 10 categories, has a greater effect on patient safety than Family as a Potentiator of Security Failures.

We can see that the participants’ statements show the strong potential of families to contribute to the safety of care and that sometimes they do so effectively. On the other hand, their contribution does not always improve safety. To change these negative contributions and to ensure that families play a role in improving patient safety, the participants identified the need for greater availability of time to intervene and work with families, which they refer to as scarce in their respective realities.

## 5. Conclusions

The results of this study are based on the perceptions of the participating nurses, whose data were processed using a scientific methodology.

With these results, it is possible to verify that the involvement of families in patient safety, in addition to presenting strong potential, adds complexity to the provision of care. This is evidenced by the higher number of categories identified in the Family as a Security Barrier compared to the lower number in the Family as a Potentiator of Security Failures.

In addition to greater complexity, the participants noted that it is necessary to intervene with and for families so that their involvement is safe, which creates a demand for the availability of higher nursing ratios than those currently in place.

This study has a limitation in that it was developed during the COVID-19 pandemic period, so it was not possible to conclude whether there were changes in the results for this reason.

In terms of the family’s potential role in the risk-management process, further studies are needed on the effectiveness of family involvement in safe care and to identify the most effective concrete strategies. It is therefore suggested that this study be replicated in other care contexts and cultural realities.

## Data Availability

The data are not publicly available due to ethical issues of respecting the anonymity of the participants and institutions involved.
